# The Effect of the Distribution Head Tilt and Diffuser Variants on the Evenness of Sowing Rye and Oat Seeds with a Pneumatic Seed Drill

**DOI:** 10.3390/ma13133000

**Published:** 2020-07-06

**Authors:** Łukasz Gierz, Piotr Markowski

**Affiliations:** 1 Faculty of Civil and Transport Engineering, Poznań University of Technology, ul. Piotrowo 3, 60-965 Poznań, Poland; 2 Department of Heavy-Duty Machines and Research Methodology, University of Warmia and Mazury, ul. Oczapowskiego 11, 10-719 Olsztyn, Poland; piotr.markowski@uwm.edu.pl

**Keywords:** pneumatic seed drill, rapid prototyping, distribution head, diffuser, diffuser variant, distribution accuracy

## Abstract

Due to the sustainable development of agriculture machines with large working widths of 4–6 m or even 9–12 m are increasingly often used for agrotechnical operations. The sowing machinery whose working widths are much bigger than the width of the seed box is equipped with a pneumatic system for transporting seeds from the seed box to coulters. One of the structural elements that affect the sowing accuracy in such seed drills is the distribution head with a diffuser. This article is about research on the influence of the distribution head deviation from the vertical position and constructional variants of the diffuser (the number of diffusion rings and the configuration of their position in the diffuser pipe, which is the distance between them) on the accuracy of distribution of a stream of rye and oat seeds (a seed-and-air stream), which differ in physical characteristics. The main elements, i.e., the innovative stream distributor in the head and the diffusion rings were made using an original design and the rapid prototyping method. The research proved that a change of 0–10° in the angle of the distribution head deviation from the vertical position significantly affected the sowing quality of oat seeds only. The position (density) of the diffusion rings in the lower section of the diffuser (near the supply elbow) was the most effective for both oat and rye seeds, where the average values of the coefficient of variation were 5.31% and 4.62%, respectively. The research results can be used to redesign the construction of the diffuser of the seed drill distribution head so as to reduce the resistance of transport of the seed-and-air mixture in order to improve seed sowing evenness.

## 1. Introduction

Sowing is one of the most important agrotechnical procedures in the cultivation of cereals and other crops. Machines with large working widths (4–6 m or even 9–12 m) are increasingly often used for sowing. These are usually pneumatic seed drills with the main central air system transporting seeds from the seed box to seed ducts ended with coulters [1]. The seed stream is distributed into the seed ducts by distribution heads [1]. In practice, vertical and horizontal distribution devices are used. In horizontal distribution devices, e.g., manufactured by Morris [2] and Chervona Zirka Elvorti [3], the controlling element is installed on the bottom of the pipe. According to the manufacturers, this should ensure effective control of the distribution of the seed-and-air stream because grains move on the bottom of the pipe under the influence of gravity. Unfortunately, in practice, this construction has not effectively solved the problem of even distribution of seeds [4,5]. Vertical devices have been more widely applied in pneumatic seed drills to distribute the seed-and-air stream. Such solutions are used in seed drills manufactured by Amazone, John Deere, Bednar and Akpil. These heads consume less power than horizontal heads to generate an airstream [6]. The linear location of the pipes facilitates their access to the coulters, which results in better energy efficiency [7]. In vertical heads, the seed-and-air stream goes through the elbow and diffuser to the distribution head. An ideally designed distribution head should symmetrically divide the main stream of seeds into smaller streams, which should go through the seed ducts to the coulters. The first pneumatic seed drill with a vertical distribution device was designed by Weist in the 1960s [8]. The device had a diffuser with a smooth internal surface, and the coefficient of variation (*CV*) ranged from 15% to 27% [5,8]. In order to improve the evenness of seed distribution, a corrugated diffuser was used. This resulted in additional turbulence of the seed-and-air stream in the pipe, which significantly improved the seed sowing evenness, where the value of the coefficient of variation (*CV*) decreased to 4–8% [4]. Apart from the diffuser and distribution head, the inlet elbow also negatively affects the sowing quality, i.e., even distribution of the seed stream. In order to eliminate the negative influence of the elbow on the evenness of distribution of the seed stream Piping [9] proposed the use of several opposing input elbows. In consequence, seed sowing evenness improved and the value of the seed sowing coefficient of variation (*CV*) was reduced.

As it is difficult to evenly distribute the seed stream in pneumatic seed drills, there is a wide range of publications about the causes of unevenness [7,9,10,11,12,13,14,15] as well as methods and apparatuses used to assess seed sowing evenness [16,17]. There have been publications concerning numerical investigations of the movement of grain mixtures and the airstream escaping from dosing units [18,19], and the distribution of the seed-and-air stream in the distribution head [20,21,22]. Unfortunately, these investigations did not allow for the even distribution of the seed-and-air stream when the distribution head was tilted from the vertical position (tilt of the head) while sowing seeds on slopes. Nor did the investigations allow for different variants of the position of diffusion rings in the diffuser (the number and distance between the rings). These are important aspects of operation of the seed drill. Researchers in India investigated the influence of the shape of the distribution head and the velocity of the airstream at the outlet on the seed distribution evenness [23]. The authors proved that the seed sowing evenness was influenced by the velocity of the airstream transporting seeds as well as the dose and physical properties of seeds. Lei et al. [24] studied the influence of the shape of the head and the velocity of the airstream transporting seeds. They found that the seed distribution evenness and energy consumption had improved at airstream velocities of 24–28 m/s for rapeseeds and 20–24 m/s for wheat seeds. The main focus of the aforementioned studies was only to determine the influence of the shape of the distribution heads on the evenness of distribution of the seed-and-air stream. However, it can be hypothesized that the evenness of distribution of the seed stream in the head is mainly affected by the vertical diffuser tube with annular corrugations (diffusion rings) forcing the dispersion of the seed-and-air stream at the cross-section of the tube and by the angle of the distribution head deviation from the vertical position. Therefore, the question arises whether annular corrugations along the entire length of the diffuser tube are necessary (they are used in seed drills by standard) or perhaps it would be more effective to use fewer diffusion rings in the right positions in the diffuser tube (an innovative solution).

There are numerous interesting technical solutions concerning distribution heads in the world patent database [25,26,27]. There are designs of distribution heads with internal guides in the form of an inner cone or other elements directing the seed-and-air stream [28,29]. However, the effect of these constructions on the evenness of distribution of the seed-and-air stream cannot be assessed without tests.

The authors of numerous studies on this subject indicate various causes of the uneven distribution of the seed-and-air mixture in the distribution head of the pneumatic seed drill [30]. The geometry of the distribution head is a typical cause [23]. There are more sophisticated explanations of the problem such as a higher concentration of seeds at the wall of the duct supplying seeds to the distribution head due to inlet elbows [31] or gravity [32]. An even distribution of the seed stream is only possible if the seed stream is distributed symmetrically. This means that the stream of seeds at the cross-section of the diffuser tube feeding the distribution head needs to be evenly distributed to all the seed ducts. In order to ensure an even distribution of seeds along the entire cross-section of the duct, a diffuser is placed in the head before the seed stream distributor. In a classic pneumatic seed drill designed according to the Weiste patent, the diffuser is a tube with wavy corrugations [8]. There are also other solutions described in the patent literature, e.g., in the form of conical protrusions or other elements [28,29]. However, the authors of these solutions did not write how the diffuser geometry affected the value of the seed sowing coefficient of variation (*CV*) and how the value of the coefficient of variation *(CV)* was affected by the elbow placed in front of the diffuser. Kravtsov et al. [33] proved that it was necessary to design a new segment of the pipeline with additional elements of the construction, which would direct the flow of the seed-and-air mixture above the elbow to limit its effect on the value of the coefficient of variation (*CV*).

Our review of the designs and tests of components of the sowing system of universal pneumatic seed drills showed that the research results presented in reference publications were not unequivocal and did not exhaust the issue under study. The problem of even distribution of the seed-and-air stream has not been solved, especially when seeds are sown on soils with different terrain. Additionally, reference publications do not provide a clear explanation of how the position of diffusion rings along the length of the diffuser tube affects the seed sowing evenness.

In view of the abovementioned facts, the aim of this study was to determine how the deviation of the distribution head from the vertical position and diffuser variants (the number of diffusion rings and the configuration of their position in the diffuser tube: the distance between them) affected the accuracy of distribution of a stream of rye and oat seeds, which differ in physical characteristics. The study was conducted to find the best configuration of distribution heads.

## 2. Materials and Methods 

There was one stage of laboratory investigations on rye and oat seeds. The mass of seeds distributed in the head of the pneumatic seed drill was measured at the research facility described in Section 2.2. The object of the study is described in Section 2.1.

### 2.1. Innovative Distribution Head

**Figure 1:**
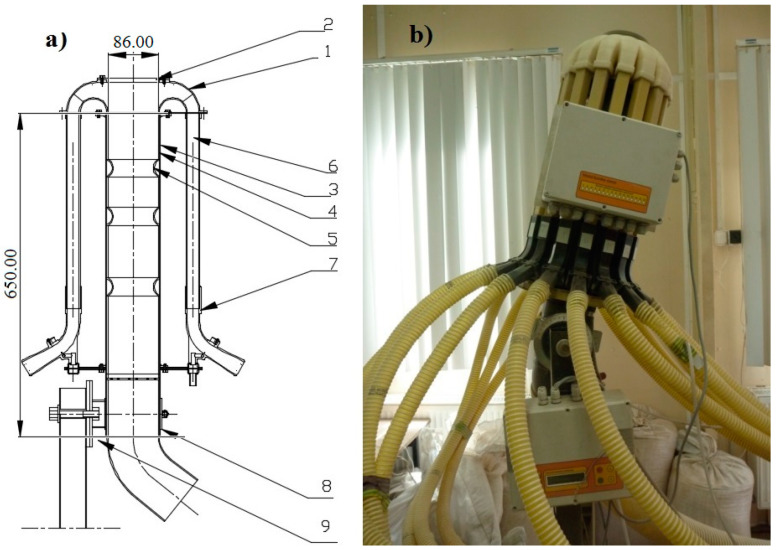
The distribution head and the diffuser of the pneumatic seed drill: (**a**) diagram with basic dimensions: 1—stream distributor, 2—cover, 3—diffuser with diffusion rings, 4—spacer sleeve, 5—diffusion ring, 6—downpipe, 7—downpipe elbow, 8—feeding pipe elbow, 9—tilting stand; (**b**) view of the prototype.

**Figure 2:**
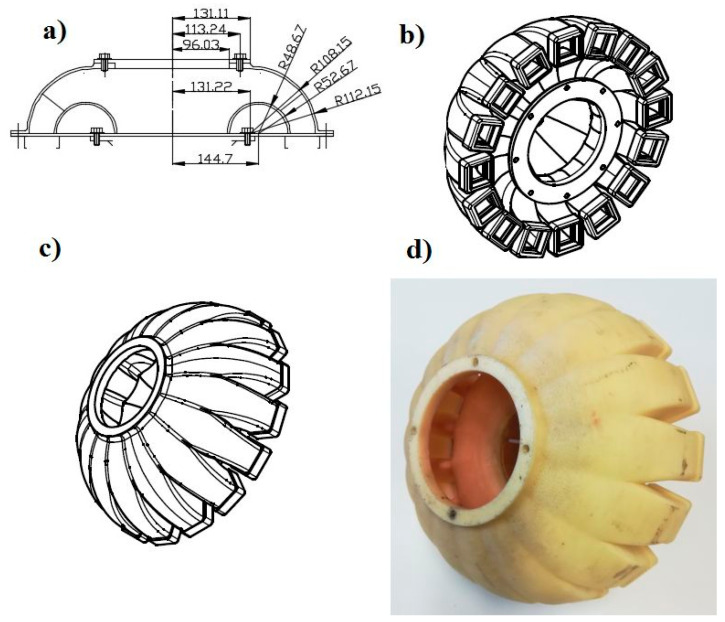
An innovative distributor of the seed-and-air stream in a pneumatic seed drill: (**a**) cross-sectional view of the stream diffuser with basic dimensions, (**b**) bottom view of the CAD 3D model, (**c**) top view of the CAD 3D model, (**d**) view of the stream diffuser prototype made with the rapid prototyping method.

**Figure 3:**
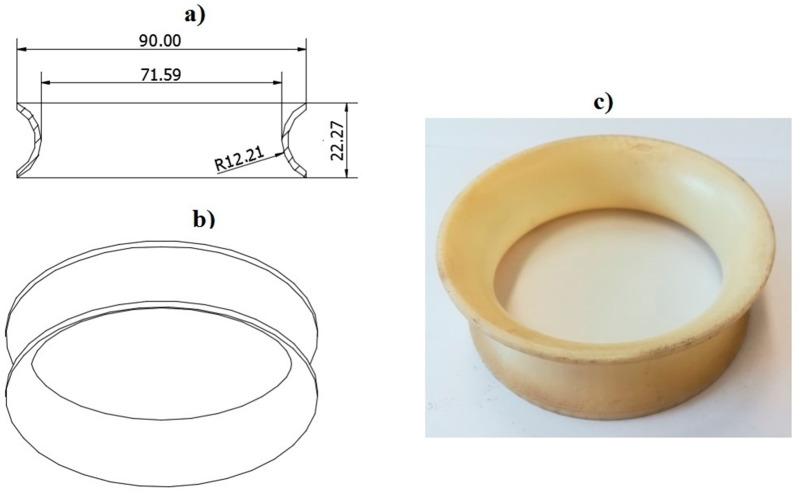
A diffusion ring of the diffuser: (**a**) cross-sectional view of the ring with basic dimensions, (**b**) a CAD 3D model of the diffusion ring, (**c**) view of the diffusion ring prototype with the rapid prototyping method.

The research object was an innovative head distributing the stream of seeds (Figure 1). It consisted of a seed stream distributor (1) with a cover (2) attached to a diffuser with diffusion rings (3). The stream distributor (Figure 2) and the diffusion rings (Figure 3) were made using an original design and the rapid prototyping method. The diffuser (3) located before the stream distributor was designed to diffuse seeds with any number of diffusion rings positioned at a set distance from each other. The diffuser was made from a PVC (Polyvinyl chloride) pipe (external diameter 90 mm, internal diameter 86 mm, length 650 mm, Figure 1), which is the outer shell (supporting structure), diffusion rings (ring rib rounding radius 7.2 mm) and spacer sleeves in the form of a PVC tube with lengths of: 60, 70, 116 and 166 mm. The dimensions of spacer rings (rib rounding radius) were selected in pilot (initial) tests, in which the dimension used in commercial solutions, i.e., about 2.4 mm, was assumed as the basic dimension and its double and triple values, i.e., 4.8 mm and 7.2 mm. The effect of the seed-and-air stream turbulence determined the selection of the rib rounding radius of 7.2 mm for specific tests. According to Gierz and Kęska [30], such a flow ensures a more even distribution of the seed stream because it is more likely that the seeds will appear at any point at the cross-section of the connector supplying the distribution head. Figure 1 shows a diagram of the distribution head with the diffuser, basic dimensions (Figure 1a) and a prototype of the distribution head with the diffuser (Figure 1b). Figure 2 shows the stream distributor: a diagram with basic dimensions (Figure 2a), a CAD 3D model (bottom view, Figure 2b), CAD 3D model (top view, Figure 2c) and a view of the stream distributor prototype made by rapid prototyping (Figure 2d). Figure 3 shows the diffusion ring: a diagram and basic dimensions (Figure 3a), a 3D CAD model (Figure 3b) and a view of the diffusion ring prototype made by rapid prototyping (Figure 3c).

During the tests, the distribution head with the diffuser was placed on a tilting stand (9) allowing the head to be tilted from the vertical position within ‒20° to 20°. The seed-and-air stream in the innovative distribution head was supplied through the feeding pipe elbow (8), diffuser (3) with diffusion rings (5) separated by spacer sleeves (4). The seed stream was divided into 16 individual streams in the distribution head. Then, it was directed to the coulters through the downpipes (6) and downpipe elbows (7) with attached pneumatic ducts.

**Figure 4:**
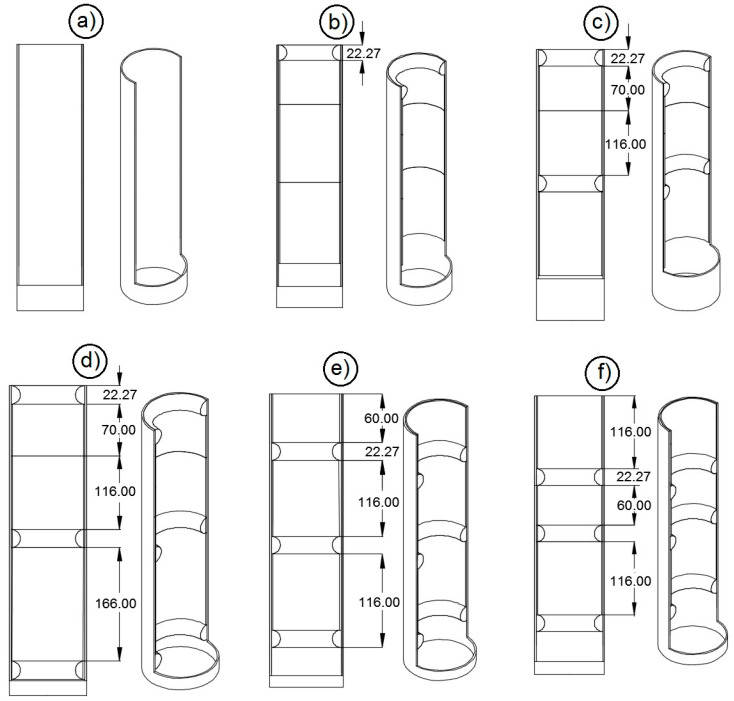
Variants of the position of diffusion rings separated by diffuser spacer sleeves—configurations used in laboratory investigations. (**a**) smooth surface; (**b**) one diffusion ring; (**c**) two diffusion rings; (**d**) three diffusion rings with a 166 mm spacer sleeve at the bottom; (**e**) three diffusion rings with a 60 mm spacer sleeve on top; (**f**) three diffusion rings with a 116 mm spacer sleeve on top.

Six variants of the diffuser shown in Figure 4 were used at the test facility between the feeding pipe elbow and the distribution head. In the first variant, a smooth-surface diffuser without diffusion rings was installed (Figure 4a). In the second variant, there was one diffusion ring at the top, immediately before the connection with the distribution head (Figure 4b). In the third variant, there was an additional diffusion ring, which was separated from the ring situated on the top of the diffuser with two spacer sleeves with a total height of 186 mm (Figure 4c). In the other three variants, three diffusion rings positioned in three different configurations shown in Figure 4d–f were used in the diffuser. In the first configuration (the fourth variant of the diffuser (Figure 4d)), the additional ring mounted on the top of the diffuser was separated from the middle ring by a spacer sleeve, which was 166 mm high (Figure 4d). The sleeves used in this configuration divided the diffuser into two almost symmetrical zones. In the second ring spacing configuration (the fifth variant of the diffuser (Figure 4e)) a 60 mm high spacer sleeve was placed as the first element from the top of the diffuser. Then, there was a diffusion ring followed by a 116 mm high spacer sleeve, then the second diffusion ring followed by a 116 mm high spacer sleeve and then the third ring (Figure 4e). The configuration also resulted in a division into two symmetrical zones but they were shifted 60 mm down. In the sixth variant (Figure 4f) there were also three diffusion rings. There was a 116 mm high spacer sleeve on the top of the diffuser, followed by the first diffusion ring and a 60 mm high spacer sleeve, then the second diffusion ring and another 116 mm high spacer sleeve and then the third diffusion ring (Figure 4f). In this configuration, the first ring was moved 116 mm away from the top. The division resulted in zones at a third and two-thirds of the distance. The height of the diffusion rings and their position in the diffuser were selected on the basis of preliminary research.

### 2.2. Test Facility

**Figure 5:**
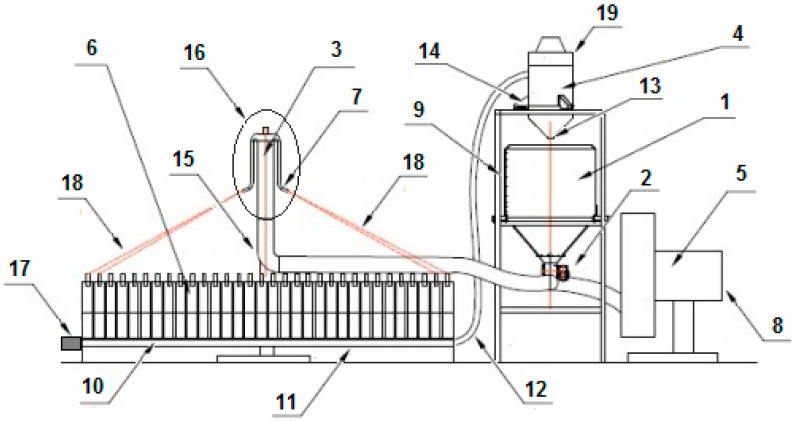
Diagram of the laboratory facility: 1—main tank, 2—sowing unit, 3—distribution head, 4—block of the automatic system weighing seeds sown, 5—main fan, 6—collecting box, 7—downpipe elbow, 8—fan motor, 9—main frame, 10—collecting box chambers, 11—collecting duct (collector), 12—suction duct, 13—closing flap, 14—strain gauge, 15—supply elbow, 16—diffuser with diffusion rings, 17—servomotor powering the cam mechanism, 18—seed ducts, 19—suction fan.

**Figure 6:**
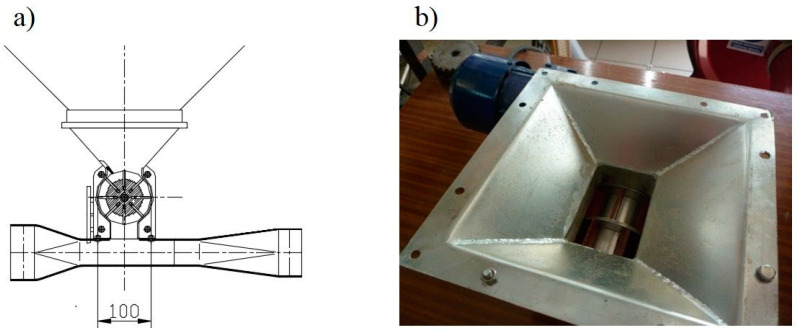
Seed dispensing unit (**a**) diagram, (**b**) prototype view.

The research on the innovative distribution head of the pneumatic seed drill was conducted at a facility that was specially designed and constructed for laboratory investigations at the Department of Heavy-Duty Machines, Poznań University of Technology, Poland (Figure 5). The test facility consisted of the main frame (9), the main seed tank (1) with a strain gauge (weighing accuracy: 0.01 kg), a sowing unit (2, diameter: 100 mm, Figure 6), the main fan generating an airstream (5) at a flow velocity of 5–40 m/s, a discharge pressure of 6 kPa and capacity of 0.680 m^3^/s, powered by a 5.5 kW AC motor (8), seed ducts (18) receiving seeds from the outlets of downpipe elbows (7) to individual chambers (6) of the collecting box (10). Before each measurement cycle, the airflow velocity was monitored with a Testo 440 meter with a hot wire probe (measurement accuracy: +/‒ 0.03 + 4% of the measured value). The airstream velocity was constant, i.e., 15.5 m/s. It was measured before the fan and calculated using the flow continuity equation to obtain an airstream velocity of 35.3 m/s at the fan outlet. The test facility was also equipped with a block of an automatic system weighing the seeds sown (4) from 16 individual chambers of the seed collecting box. It also had a computer with dedicated software for data acquisition and calculation of the value of the transverse sowing unevenness index, which was based on the dependence used for the calculation of the coefficient of variation (*CV*).

Seeds of two cereal species were used in the research: rye (Dańkowskie Rubin cultivar, Choryń, Poland) with a thousand kernel weight of 34.1 g ± 0.83 and a moisture content of 8.5% and oats (Spartan cultivar) with a thousand kernel weight of 32.2 g ± 0.62 and a moisture content of 9.3%. These were certified seeds purchased from Poznańska Centrala Nasienna (Poznań, Poland).

At the end of each seed sowing test cycle (trial) at the test facility, the 16 chambers of the seed collecting box were opened sequentially and their contents were weighed and returned in a closed circuit to the main tank (1). Therefore, valves opened by means of a cam mechanism were installed at the bottom of the chambers of the collecting box. The valve opening mechanism consisted of a movable cam carriage driven by a BG 65X25 SI Dunkermotoren servomotor (17) by means of a toothed belt (10). Borland Pascal—a specially written program in the RAD Delphi 2010 environment (Embarcadelo) was used to control the servomotor (17). A rectangular collector (11) was mounted under the valves along the 16-chamber collecting box (6). When the valves opened, seeds fell into the collector and then they were sucked into the airstream generated by the suction fan (19). Then, the seeds went through the suction duct (12) to the separating cyclone located above the main tank (1). The conical bottom of the separating cyclone in the lower part was closed by a flap (13), which was not connected to the cylindrical part of the cyclone. It was based on three strain gauges (14) for weighing seeds from each chamber of the collecting box separately with an accuracy of 0.005 kg. During the transport of seeds from the chambers of the collecting box (10) the cyclone flap (13) was closed with a force of about 500 N by the negative pressure in the system. Then, the suction blower drive (19) was switched off and the bottom of the cyclone fell on the strain gauges. When the flap closing (13) the bottom of the cyclone fell, it was electromagnetically locked by the control system. The entire seed weighing cycle was fully automated. After the seeds from all the 16 chambers of the collecting box (10) were weighed, a test report was generated and the results were saved in *. txt files.

The measuring cycle at the laboratory facility looked as follows:

1. Measurement parameters were read from the assumed research program: airstream velocity and measurement time;
2. The motor of the main fan was switched on. It was necessary to wait until its rotational speed was constant;
3. The cam carriage was checked to ensure that it was in its extreme position;
4. The rotational speed of the fan shaft was adjusted to achieve the assumed airstream velocity at the inlet of the seed dosing unit;
5. The sowing unit was started and the desired rotational speed of the sowing shaft was adjusted;
6. When the unit reached the assumed efficiency, it was necessary to wait for 5 s to determine the sowing conditions;
7. The seed-and-air stream was directed to the distribution head;
8. Seeds were sown according to the assumed research plan with a measurement time about 10–60 s;
9. The drive of the sowing unit and the main fan were switched off;
10. The recirculation unit motor fan was started;
11. The negative pressure in the cyclone was checked;
12. The valve of another chamber in the box was opened;
13. After 30 s, the recirculation unit motor was turned off;
14. The weight of seeds sown and the weight of the whole seed tank were read from the force sensors;
15. The flap in the lower part of the cyclone was opened and the seeds were released;
16. When the valve cam carriage reached its extreme position, Chamber 1 was the starting point;
17. The carriage moved to the starting position;
18. The measurement results were processed and saved in the database (the weight of the seeds from all the chambers was saved in a text file; the average weight of seeds, standard deviation and coefficient of variation (*CV*) were calculated).

### 2.3. Laboratory Facility Control System

**Figure 7:**
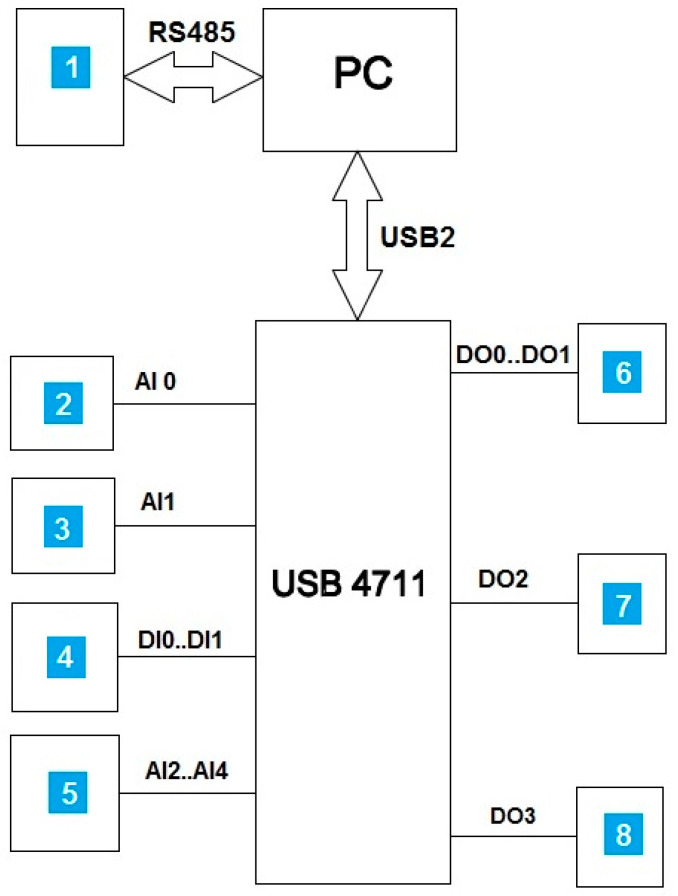
Block diagram of the laboratory control system: 1—seed counting unit, 2—seed weighing unit, 3—weighing unit of the main tank, 4—carriage position sensor, 5—pressure sensor, 6—carriage drive servomotor, 7—fan drive, 8—sowing plant drive.

**Figure 8:**
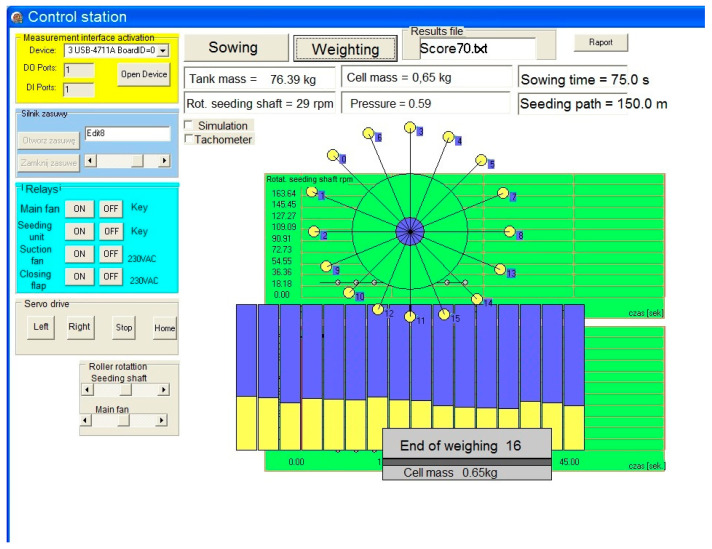
A dialogue form of the program controlling the measuring facility.

The most important element of the test facility is the original control system with a dedicated program created in the Embarcadero RAD Delphi 2010 environment. The program, which works under the Windows operating system, uses measurement libraries provided by Advantech and servomotor manufacturers, written in the Delphi 2010 RAD studio environment. The computer program communicates with the environment through the Advantech USB 4711A measuring interface and additional coupling systems, including electromagnetic relays and opto-isolators. The program also uses its own graphic and mathematical library grafika_v3 as a supplement to the control system. The control system uses: serial transmission channels (USB2), four digital outputs (DO0–DO3), two digital inputs (DI0, DI1) and four analog inputs (AI0–AI4, Figure 7). Data and results are displayed in a dialogue form (Figure 8) and saved in files for further statistical analysis. The software sends voice messages. It is an advantage because the operating staff does not need to check the progress on the monitor. The program operates as follows: on startup, the main dialogue form appears (Figure 8), where the test parameters should be entered. When the button is pressed, the sowing test at the set time starts. The sowing time results from the length of the measuring section assumed in the test program and from the assumed working speed of the seed drill unit. When the first procedure is completed, the fully automated weighing procedure starts under the control of the program.

### 2.4. Indicators and Calculations

**Figure 9:**
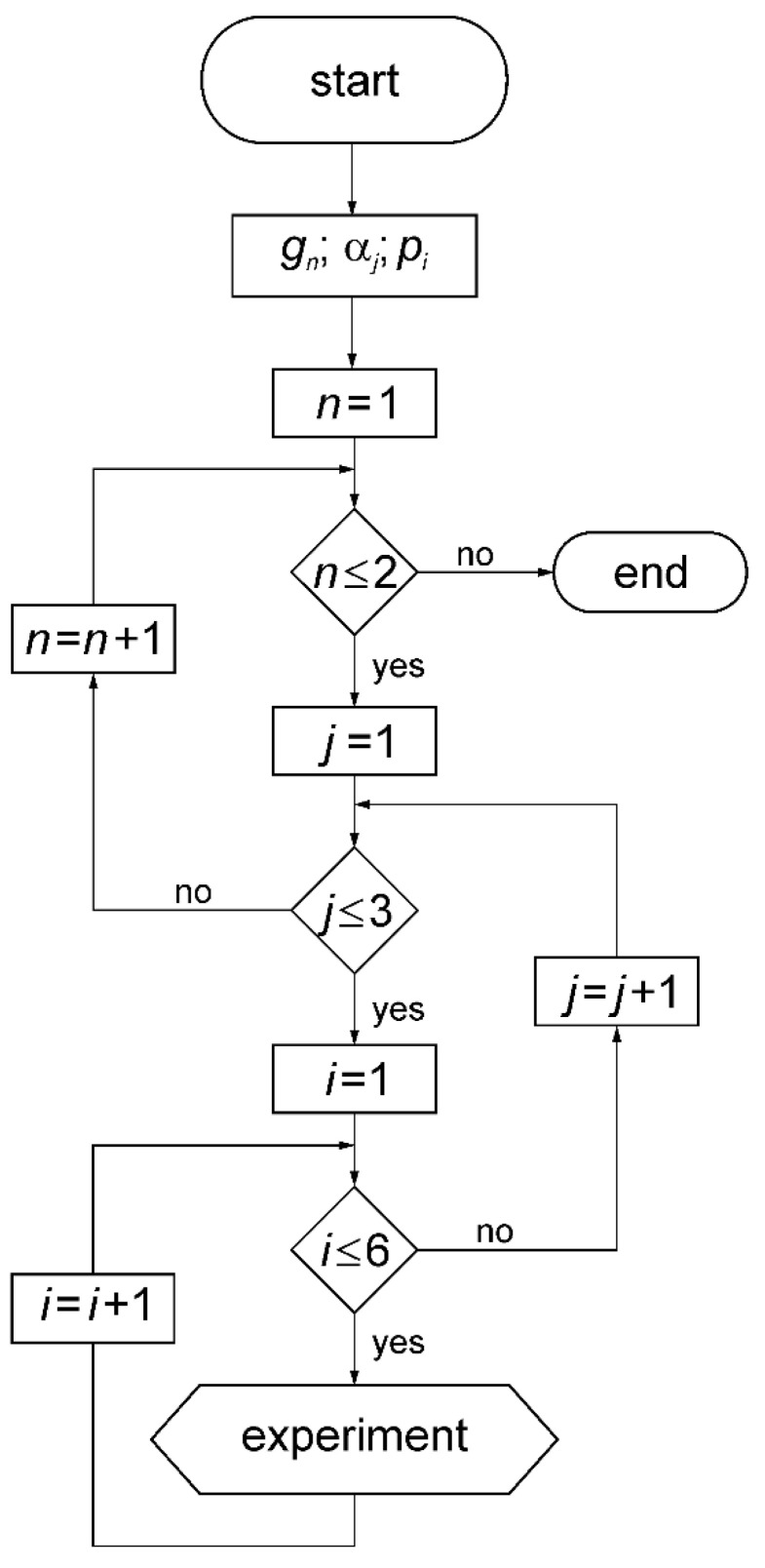
The algorithm used for measurements of the transverse rye and oat seed sowing evenness: *n, j, i*—consecutive numbers (values) of variable parameters (*g, α, p).*

There were four replicates of the experiment for all combinations of variable parameters according to the algorithm shown in Figure 9.

The following factors were assumed in laboratory investigations conducted on both seed species (rye and oats):

(a) Constant:

− Theoretical (assumed) sowing speed—2 m/s;
− Sowing shaft rotational speed—29 rpm;
− Assumed length of the measuring section (sowing pathway)—150 m;
− Airflow velocity at the fan inlet 15.5 m/s.

(b) Variable:

− The angle of the distribution head tilt from the vertical position *α_j_*: 0°, 5° and 10°;
− Diffuser variant: the position and number of diffusion rings *p_i_*: *p*_1_, *p*_2_, *p*_3_, *p*_4_, *p*_5_, *p*_6_;
− Seed species *g_n_*: rye, oats.

(c) Resulting factors:

− Coefficient of variation (*CV*).

The influence of the angle of the distribution head deviation from the vertical position and the deflector variant on the evenness of distribution of the seed-and-air stream to individual coulters was determined by calculating the coefficient of variation (*CV*) from the dependence shown below [34,35], in accordance with ISO 7256/2:

(1) $$CV=\frac{S}{X}\times 100\%$$

where *S* is the standard deviation of the average weight of seeds sown in four replicates and *X* is the average weight of seeds collected from all coulters.

### 2.5. Statistical Analysis

The results of measurements of the coefficient of variation (*CV*) in the sowing of oat and rye seeds, depending on the angle of the distribution head deviation from the vertical position (*α_i_*) and the diffuser variant—the configuration and number of diffusion rings (*p_i_*) were analyzed statistically, including analysis of variance (ANOVA). The aim of the analysis was to compare the average values of the coefficient of variation (*CV*), which was used as a sowing evenness indicator, at different combinations of independent variables (*α_i_*) and (*p_i_*). If there was a statistically significant difference between the average values of the coefficient of variation, post hoc tests were carried out. The Statistica v. 13 PL software package was used for calculations. The level of significance α = 0.05 was assumed in the analysis and inference.

## 3. Results and Discussion

When assessing the configurations of the diffuser (*p_i_*) and the distribution head angle of deviation from the vertical position (*α_i_*) it was important to answer the question of whether the seed sowing evenness measured with the coefficient of variation (*CV*) did not differ significantly depending on the assumed parameters of independent variables, i.e., the position and number of diffusion rings (*p_i_*) and the three angles of the distribution head deviation from the vertical position (*α_i_*). Therefore, analysis of variance (ANOVA) was applied to consider the null hypotheses H_0_ that the mean coefficients of variation (*CV*) were equal and the alternative hypotheses H_1_ that the mean coefficients of variation were not equal. The significance level of the statistical analysis was α = 0.05.

As far as the oat seeds are concerned, the null hypothesis H_0_ should be rejected in favor of alternative hypothesis H_1_ (Table 1). As a result of the analysis of the data in Table 1, the average values of the coefficient of variation (*CV*) in the vertical distribution head variant were significantly different from the average values in the variant where the head was tilted from the vertical position at an angle of 10°. The analysis showed that when the head deviated from the vertical position at an angle of 10°, the seed sowing evenness (even distribution of the seed-and-air stream in the distribution head) deteriorated and the value of the coefficient of variation (*CV*) increased by almost 31% from 10.36% (the vertical position of the distribution head) to 13.55% (the distribution head tilted from the vertical position at 10°). Yatskul et al. [35] observed a similar dependence while sowing wheat seeds and tilting the distribution head within 0–22°.

However, the analysis of variance for the rye seeds showed that there were no grounds to reject the null hypothesis H_0_, according to which the value of the coefficient of variation for the first independent variable assumed in the study (the angle of the distribution head deviation from the vertical position *α_i_*) was equal (Table 2).

**Figure 10:**
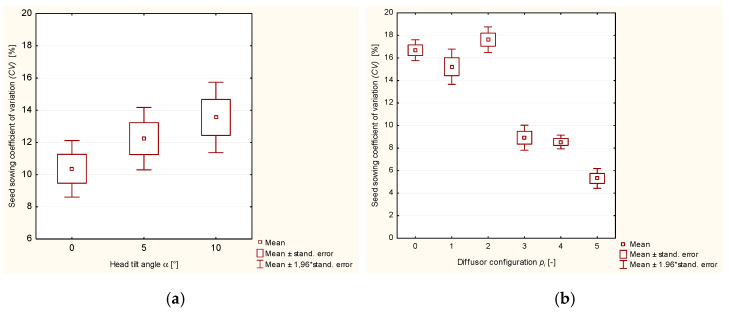
Variability of the coefficient of variation (*CV*) for oat seeds sowing evenness depending on: (**a**) the head tilt angle, (**b**) the diffuser variant (the configuration and number of diffusion rings).

As far as the second independent variable is concerned, i.e., the diffuser variant (the position and number of diffusion rings, *p*_i_), the analysis of variance showed that null hypotheses H_0_ about equal values of the mean coefficient of variation (*CV*) must be rejected in favor of alternative hypotheses H_1_ for both oat and rye seeds. Even when only one diffusion ring was used in the diffuser tube, both the rye and oat seeds were sown more evenly than in the diffuser with a smooth internal surface. The research results were compatible with the results of tests conducted on wheat seeds by Gierz and Kęska [30] and Yatskul et al. [35]. For both oat and rye seeds, the lowest mean values of the coefficient of variation were noted for variants *p*_4_–*p*_6_ with three diffusion rings in different configurations (Figure 4d,f). The analysis of the test results showed that the position (congestion) of diffusion rings in the lower part of the diffuser was the most effective (test variant *p*_6_). This regularity was observed when both rye and oat seeds were sown. The average values of the coefficient of variation were 5.31% and 4.62%, respectively. As was expected, the distribution of the seed-and-air stream in the head with a smooth tube (variant *p*_1_) resulted in considerable seed sowing unevenness. The average values of the coefficient of variation (*CV*) were 16.68% for oat seeds and 15.94% for rye seeds (Table 1 and Table 2). The values of the coefficient of variation (*CV*) also showed considerable sowing unevenness, which negatively affects the yield, as was indicated in reference publications [36,37]. As a result of the data concerning oat seeds sowing evenness in Table 1 and Figure 10, three homogeneous groups can be distinguished (in terms of differences between the mean values) for the second independent variable, i.e., the diffuser variant. The first group includes the results of seed sowing measurements for variants *p*_1_-p_3_ with a smooth inner surface of the diffuser tube and with one or two diffusion rings (Figure 4a–c). The second group includes the results of measurements when the seeds were sown with a diffuser with three diffusion rings positioned in two configurations, variants *p*_4_ and *p*_5_ (Figure 4d,e). The third group includes the results of the seed sowing variation coefficient where the diffuser had three rings positioned in configuration *p*_6_ (Figure 4f).

**Figure 11:**
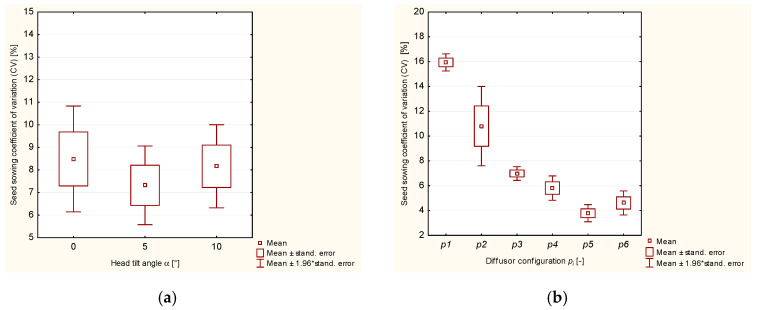
Variability of the coefficient of variation (CV) for rye seeds sowing evenness depending on: (**a**) the head tilt angle, (**b**) the diffuser variant (the configuration and number of diffusion rings)

For greater clarity, the results of the analysis of variance in Table 1 and Table 2 are illustrated in Figure 10 and Figure 11.

Further statistical analysis involved answering the question of whether the mean values of the coefficient of variation (*CV*) differed significantly depending on the seed species. One-way analysis of variance (ANOVA) was applied with null hypothesis H_0_ that the mean values of the coefficient of variation (*CV*) were equal and alternative hypothesis H_1_ that the mean values of the coefficient of variation were not equal. The analysis of variance (Table 3) showed that hypothesis H_0_ about the equality of the mean values of the coefficient of variation should be rejected in favor of alternative hypothesis H_1_ about the lack of equality of the mean values of the coefficient of variation. As a result of the data in Table 3, the mean value of the coefficient of variation (*CV*) for rye seeds (*CV* = 7.99%) was significantly lower, i.e., by nearly 34% (tatistically significant difference at a significance level α = 0.01) than the mean value of the coefficient of variation for oat seeds (*CV* = 12.05%).

**Figure 12:**
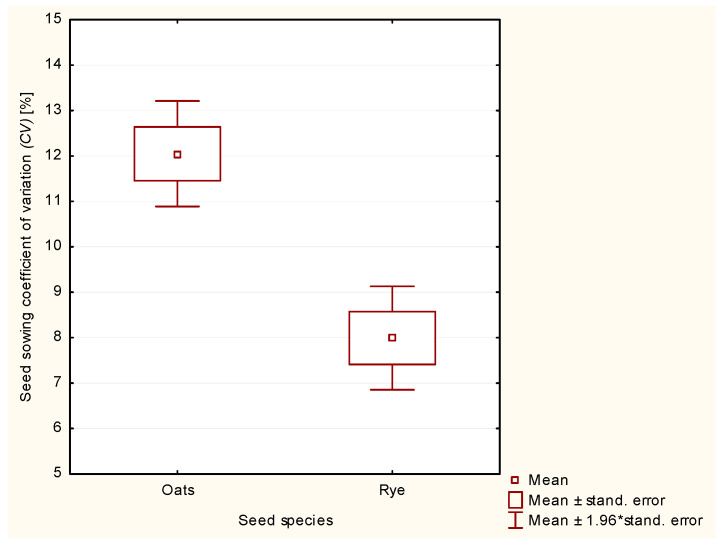
The values of the coefficient of variation *(CV)* for sowing evenness depending on the seed species.

Like the previous statistical analysis, the results of the analysis of variance shown in Table 3 are also illustrated in Figure 12 for greater clarity.

## 4. Conclusions

In the last 20–30 years there has been dynamic development in the construction of farming machinery. It has been observed not only in systems supervising and controlling the parameters of operation of machines but also in new constructions and materials used in farming machines. This trend has also been observed in sowing machinery, i.e., universal and precision seed drills. Seed drill manufacturers try to improve the qualitative parameters of sowing, i.e., the longitudinal and transverse evenness of their distribution. Although there has been some technological progress, it is still necessary to make further improvements. Engineers usually change the construction of the functional units of seed drills, which have the biggest influence on sowing evenness. In pneumatic seed drills, the sowing unit is usually altered, especially the distribution head and diffuser. The authors of this article followed this trend and investigated the influence of the distribution head deviation from the vertical position and diffuser variants (the number of diffusion rings and the configuration of their position in the diffuser tube: the distance between them) on the precision of the distribution of the stream of rye and oat seeds, which have different physical characteristics. The research showed that the angle of the seed drill tilt resulting from the terrain, which was simulated in the tests by changing the angle of the distribution head deviation from the vertical position within 0-10°, significantly affected only the oat seeds sowing quality. When the distribution head deviated from the vertical position by 10°, the seed sowing quality deteriorated. This effect was reflected by the value of the coefficient of variation (the value of the coefficient of variation changed from 10.36% at a tilt angle of 0° to 13.55% at 10°), regardless of the diffuser variant, i.e., the number and configuration of the diffusion rings. The tests conducted on the rye seeds showed that the difference between the mean values of the coefficient of variation depending on the angle of the distribution head deviation from the vertical position was not significant and the value of the coefficient of variation ranged from 7.32% to 8.49%.

As far as the second variable, i.e., the diffuser variant is concerned, the research showed that in comparison with the configuration with the smooth inner surface of the diffuser tube, the installation of one diffusion ring in the tube improved the evenness of sowing both oat and rye seeds. Further analysis of the test results showed that the positioning of the diffusion rings in the lower section of the diffuser (close to the supply elbow) was the most effective configuration (test variant *p*_6_). This regularity was observed when both oat and rye seeds were sown. The mean values of the coefficient of variation were 5.31% and 4.62%, respectively. As expected, when a smooth pipe (variant *p*_1_) was used before the head, the distribution of the seed-and-air stream resulted in high sowing unevenness. The mean values of the coefficient of variation (*CV*) for the sowing of oat and rye seeds were 16.68% and 15.94%, respectively. 

Further research should be conducted to construct a shorter diffuser and thus reduce the dimensions of the distribution head. Rapid prototyping and 3D printing technologies are the best methods for this purpose [38,39]. Therefore, it is necessary to continue work (tests) in order to determine the optimal number of rings and their distribution (distance) in the diffuser zone located right behind the supply pipe elbow.

## Figures and Tables

**Table 1:** The analysis of variance for the coefficient of variation (*CV*) in transverse oat seeds sowing evenness depending on the angle of the distribution head deviation from the vertical position α (°) and the diffuser variant *p* (the configuration and number of diffusion rings).

No.	Variable	Mean (%)	Standard Deviation (-)	Total Ranks	Mean Rank (-)	Statistical Value H (-)	*p* (-)	Significance of Differences
**Head Tilt Angle** **α (°)**								
1.	α	10.36	4.39	688.50	28.69	6.1375	0.0465	α_0_ < α_10_ **
2.	α_5_	12.24	4.85	893.00	37.21			
3.	α_10_	13.55	5.48	1046.50	43.60			
**Diffuser Variant: Configuration and Number of Diffusion Rings *p***								
1.	*p*_1_	16.68	1.63	670.00	55.83	59.3470	0.0000	*p*_1_ > *p*_4_; *p*_5_ ** p_6_ < *p*_1_; *p*_2_; *p*_3_ * p_3_ > *p*_4_; *p*_5_; *p*_6_ *
2.	*p*_2_	15.22	2.75	577.00	48.08			
3.	*p*_3_	17.62	2.00	712.00	59.33			
4.	*p*_4_	8.92	1.96	293.00	24.42			
5.	*p*_5_	8.55	1.08	282.00	23.50			
6.	*p*_6_	5.31	1.54	94.00	7.83			

* Statistically significant difference at α = 0.01; ** statistically significant difference at α = 0.05.

**Table 2:** The analysis of variance for the coefficient of variation (*CV*) in transverse rye seeds sowing evenness depending on the angle of the distribution head deviation from the vertical position α (°) and the diffuser variant *p* (the configuration and number of diffusion rings).

No.	Variable	Mean (%)	Standard Deviation (-)	Total Ranks	Mean Rank (-)	Statistical Value H (-)	*p* (-)	Significance of Differences
**Head Tilt Angle** **α (°)**								
1.	α_0_	8.49	5.86	889.00	37.04	0.9300	0.6282	No statistically significant differences
2.	α_5_	7.32	4.35	800.50	33.35			
3.	α_10_	8.16	4.60	938.50	39.10			
**Diffuser Variant: Configuration and Number of Diffusion Rings *p***								
1.	*p*_1_	15.94	1.22	776.00	64.67	55.6073	0.0000	*p*_1_ > *p*_4_; *p*_5_; *p*_6_ * p_2_ < *p*_5_ * p_2_ < *p*_6_ ** p_3_ > *p*_5_ **
2.	*p*_2_	10.81	5.65	638.00	53.17			
3.	*p*_3_	6.99	0.97	485.50	40.46			
4.	*p*_4_	5.81	1.73	351.50	29.29			
5.	*p*_5_	3.79	1.22	145.00	12.08			
6.	*p*_6_	4.62	1.71	232.00	19.33			

* Statistically significant difference at α = 0.01; ** statistically significant difference at α = 0.05.

**Table 3:** The analysis of variance of the coefficient of variation (*CV*) for sowing evenness depending on the seed species

No.	Seed Species	Mean (%)	Standard Deviation (-)	Total Ranks	Mean Rank (-)	Statistical Value H (-)	*p* (-)	Significance of Differences
1.	Oats	12.05	5.03	6477.50	89.97	25.2447	0.0000	R < O*
2.	Rye	7.99	4.94	3962.50	55.03			

* Statistically significant difference at α = 0.01; *p*.
